# Introducing the combined atlas framework for large‐scale web‐based data visualization: The GloNAF atlas of plant invasion

**DOI:** 10.1111/2041-210X.13820

**Published:** 2022-02-22

**Authors:** Sebastian C. Hancock, Franz Essl, Menno‐Jan Kraak, Wayne Dawson, Holger Kreft, Petr Pyšek, Jan Pergl, Mark van Kleunen, Patrick Weigelt, Marten Winter, Georg Gartner, Bernd Lenzner

**Affiliations:** ^1^ Research Division Cartography, Department of Geodesy and Geoinformation Vienna University of Technology Vienna Austria; ^2^ Bioinvasions, Global Change, Macroecology‐Group, Department of Botany and Biodiversity Research University Vienna Vienna Austria; ^3^ Faculty of Geoinformation Science and Earth Observation University of Twente Enschede the Netherlands; ^4^ Department of Biosciences Durham University Durham UK; ^5^ Biodiversity, Macroecology & Biogeography University of Göttingen Göttingen Germany; ^6^ Centre of Biodiversity and Sustainable Land Use University of Göttingen Göttingen Germany; ^7^ Czech Academy of Sciences Institute of Botany, Department of Invasion Ecology Průhonice Czech Republic; ^8^ Department of Ecology, Faculty of Science Charles University Prague Czech Republic; ^9^ Ecology, Department of Biology University of Konstanz Constance Germany; ^10^ Zhejiang Provincial Key Laboratory of Plant Evolutionary Ecology and Conservation Taizhou University Taizhou China; ^11^ Campus‐Institut Data Science Göttingen Germany; ^12^ German Centre for Integrative Biodiversity Research (iDiv) Halle‐Jena‐Leipzig Leipzig Germany

**Keywords:** atlas, cartography, D3, framework development, GloNAF, invasive alien species, JavaScript, web mapping, workflow

## Abstract

1. Large‐scale biodiversity data, for example, on species distribution and richness information, are being mobilized and becoming available at an increasing rate. Interactive web applications like atlases have been developed to visualize available datasets and make them accessible to a wider audience. Web mapping tools are changing rapidly, and different underlying concepts have been developed to visualize datasets at a high cartographic standard.
2. Here, we introduce the Combined Atlas Framework for the development of interactive web atlases for ecological data visualization. We combine two existing approaches: the five stages of the user‐centred design approach for web mapping applications and the three U approach for interface success.
3. Subsequently, we illustrate the use of this framework by developing the Atlas of Plant Invasions based on the Global Naturalized Alien Flora (GloNAF) database. This case study illustrates how the newly developed Combined Atlas Framework with a user‐centred design philosophy can generate measurable success through communication with the target user group, iterative prototyping and competitive analysis of other existing web mapping approaches.
4. The framework is useful in creating an atlas that employs user feedback to determine usability and utility features within an interactive atlas system. Finally, this framework will enable a better‐informed development process of future visualization and dissemination of biodiversity data through web mapping applications and interactive atlases.

## 1 INTRODUCTION

In the last decade, large amounts of biodiversity data have been mobilized, organized and compiled in global datasets (e.g. across taxa: Jetz et al., 2012; plants: van Kleunen et al., 2015, 2019, Weigelt et al., 2020; amphibians and reptiles: Capinha et al., 2017; birds: Dyer, Cassey, et al., 2017; across taxa: Pagad et al., 2018). This upsurge of available information has concomitantly led to an increase in publications on global patterns and processes of global biodiversity, including biological invasions (e.g. Capinha et al., 2017; Dawson et al., 2017; Dornelas et al., 2018; Dyer, Redding, et al., 2017; Hudson et al., 2017; Pyšek et al., 2017; van Kleunen et al., 2015) and has substantially advanced biodiversity science.

One established way of visualizing spatial data is through the development of an atlas, that is, a tool historically defined as a collection of maps that is comprehensive in its field, arranged systematically, authoritatively edited and presented in a unified format (Alonso, 1968). Modern definitions of atlases, though similar, have become more flexible regarding the medium, organization, spatial extent and content due to the emergence of technology (e.g. digital media, the world wide web, geographical information system software) within the field (Panchaud et al., 2013). The modernization of this definition for cartography has brought about many changes in the way that atlases are now conceived, produced, disseminated and used (Vozenilek, 2019).

A major dissemination pathway to increase the accessibility of large‐scale biodiversity datasets is web‐based applications (or online atlases). There has been substantial progress in the development of relevant web mapping and web geographical information systems (GIS) technologies (Farkas, 2017); thus, such tools have become more important in ecological and other biological fields (Janicki et al., 2016). A major advantage of web mapping applications is that they offer an effective way to provide geospatial information without the need for additional software (Machwitz et al., 2019). Open‐source web mapping and data visualization JavaScript libraries, such as Leaflet (Agafonkin, 2010) and data‐driven documents (D3; Bostock et al., 2011), combined with a user‐centred design approach, can lead to the successful creation of online interactive maps (Roth & Harrower, 2008).

At the same time, new challenges emerge concerning web mapping applications, such as geospatial organization, access, display and the use of maps as dynamic portals to inter‐connected, distributed, geospatial data resources (MacEachren & Kraak, 2001). Interactive web mapping applications further urge the developer to design a user interface that is relevant and intuitive in its usability to the target audience, that is, focus on the utility‐usability trade‐off of web mapping applications. This interactivity enhances the experience of using the web mapping application and allows the user to adapt the cartographic image of the data to one that matches their own view and needs (Ormeling, 1995). If this interaction process works smoothly, a web mapping application will benefit the target audience and have the desired impact.

To facilitate the future development of web mapping applications of large‐scale biodiversity data while ensuring successful and intuitive target audience usability (i.e. interface success; Roth, Ross, et al., 2015), we introduce a reusable framework for interactive atlas creation—the Combined Atlas Framework. Subsequently, we will illustrate the application of the newly developed Combined Atlas Framework by applying it to the Global Naturalized Alien Flora (GloNAF) database (van Kleunen et al., 2015, 2019) to create the ‘Atlas of Plant Invasions’, which provides a visual interface on the distribution of alien and naturalized alien plant species across national and subnational regions of the world. Through the Atlas of Plant Invasions, we highlight how scientists can increase the accessibility of large biodiversity datasets to researchers, decision‐makers and other stakeholders from an invasion science perspective. Furthermore, we assess mapping functionalities from other web mapping initiatives commonly used. Finally, we discuss the advantages of applying the Combined Atlas Framework in biodiversity research to facilitate data dissemination and simultaneously ensure the usage by the respective target audiences.

## 2 MATERIALS AND METHODS

**FIGURE 1:**
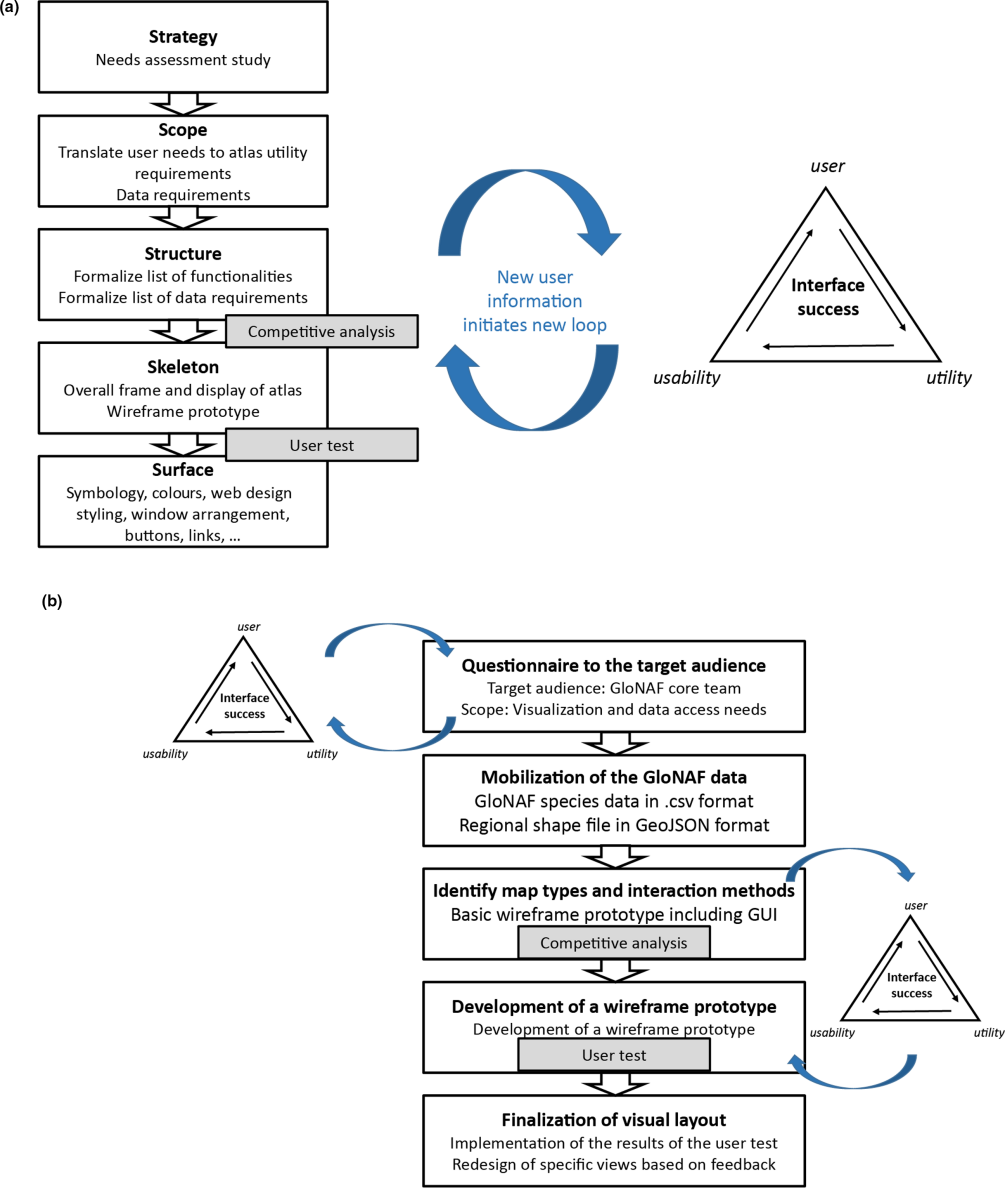
(a) The developed framework for interactive atlas creation. On the right, the triangle shows the three U approach of interface success, on the left the five stages user‐centred design is shown. Both approaches can be combined using iterative feedback loops at each and between all five stages. The competitive analysis and user test illustrate overlap between procedures undertaken at different stages and are the most important positions to initiate a feedback look. (b) Development stages and key procedures during the development of the atlas of plant invasions following the combined atlas framework

To create an interactive atlas, we propose to employ a user‐centred design (UCD) framework, which is considered essential for many web mapping projects (Tsou, 2011). In a UCD framework, web cartographers design an effective and intuitive cartographic representation by focusing on creating user interfaces, mapping functions and dynamic map content (Roth, Ross, et al., 2015). An effective web mapping application framework should be user‐centred but should also consider the utility and usability of the application (Roth, Ross, et al., 2015). The Combined Atlas Framework combines the five‐stage (strategy, scope, structure, skeleton and surface stage) UCD framework (Tsou, 2011; Tsou & Curran, 2008) with the three U (Usability, Utility and Users) approach for interface success by Roth et al. (2015). Integration of both theoretical approaches is achieved by incorporating an iterative element to the five‐stage UCD approach to gather feedback on the utility and usability of the atlas (Figure 1a). This enables the developer to better measure interface success and increases the flexibility of the process. While the user _→_ utility _→_ usability loop is most advantageous between the skeleton and surface stages, it can in practice be integrated at any of the five stages as new information gathered from the users will result in updated utility and usability purposes (Figure 1a). Additional background information to both theoretical approaches with a description of the content of each of the five stages and three U approach is found in Material S1.

We applied the Combined Atlas Framework to the GloNAF dataset (Pyšek et al., 2017; van Kleunen et al., 2019) to illustrate its usability for ecological data. GloNAF is a dynamic database that contains information on the occurrence of alien and naturalized alien vascular plant taxa across the globe. While alien taxa are those that have been introduced to regions beyond their native range due to human agency, naturalized alien taxa are those that have been reported to have established self‐sustaining populations in the wild (following the definition of Blackburn et al., 2011; Richardson et al., 2000). The database includes 13,939 taxa and covers 1029 geographical regions (countries, federal states, provinces, districts and islands) (van Kleunen et al., 2019). The data sources include naturalized alien plant compendia, national and subnational checklists published in scientific journals, as books or as Internet resources (Pyšek et al., 2017; van Kleunen et al., 2019). GloNAF does not include point occurrence information for individuals or populations of naturalized alien species and thus does not provide a high‐resolution picture of the extent of invasion within a country or subnational entity. The database is curated with frequent updates of existing and integration of new checklists, being added to the first version published in van Kleunen et al. (2019). However, the atlas described here is based on the static database version 1.2 published in van Kleunen et al. (2019). The source code for the interactive atlas is freely available online, along with the data used on github and referenced on Zenodo (Sebastian Hancock, 2022) and the views are accessible through a homepage (https://sebastian‐ch.github.io/glonafAtlas), functioning similarly to a table of contents.

Below, we outline the steps and procedures performed during the development of the Atlas of Plant Invasions following the Combined Atlas Framework. Additionally, key steps are shown in Figure 1b.

### 2.1 Strategy stage

This stage involves defining both the target users and their needs, recognizing the differences between the wide range of potential users in the field (Haklay & Zafiri, 2008).

To determine the needs of the GloNAF core team for an Atlas of Plant Invasion, a questionnaire was sent out to the target audience (i.e. the GloNAF core team consisting of 11 people), to determine their visualization needs. The questionnaire included the following four questions:

1. Is there any specific data in the GloNAF dataset that you are interested in seeing visualized? (e.g. Counts of taxa per TDWG region? Families per region? Mapping where a specific plant is invasive? Naturalized vs. alien for a region? Visualizing inventory completeness?)
2. Would a data download feature be useful?
3. If yes, I know there is a main focus on global patterns, but would a feature that lets a user download data for specific regions or taxa be useful? (e.g. If you were only interested in Hawai’i you could download that data only, or if you were only interested in one plant/taxa/family you could download just that information).
4. Would having multiple map views at the same time be useful? This would give the possibility for comparisons between different regions.

### 2.2 Scope stage

This stage involves translating what the target users want into tangible goals, establishing the scope of the interactive atlas.

Major mapping tasks for the Atlas of Invasions included interactive map manipulation, querying attributes and styling maps based on attributes. The data required from the GloNAF dataset were obtained in CSV format. A shapefile with all relevant regions based on the International Working Group on Taxonomic Databases for Plant Sciences (TDWG) classification (Brummit 2001) was obtained and converted into a GeoJSON file, which is the standard format for web mapping procedures. Finally, a global GeoJSON file of all countries was obtained from Natural Earth data, an open‐source storehouse supported by the North American Cartographic Information Society.

### 2.3 Structure stage

This stage involves the formalization of the mapping functionalities of the atlas by creating a list of tools needed in the atlas.

As part of the structure stage in the development of the Atlas of Plant Invasions, we performed a competitive analysis. We assessed a set of available biodiversity web mapping platforms and online atlases to gather information on their specific strengths and weaknesses, and methods. We selected a set of online atlases, though not all of them self‐define as atlases, with different scopes (Table 1): the Map of Life (Jetz et al., 2012), Ant Maps (Janicki et al., 2016), the Global Inventory of Floras and Traits—GIFT (Weigelt et al., 2020), the Allen Coral Atlas (Allen Coral Atlas, 2020), the Atlas of Biodiversity Conservation in the Coral Triangle (Asaad et al., 2019), the Fish Atlas of Germany and Austria (Brunken & Vatterrott, 2020) and the Gender Atlas of Austria (Wenk et al., 2015). The assessment aimed to identify map types and interaction methods commonly used in comparable projects and gain insights into patterns or similarities between the functionalities. For comparison purposes, we measured representation methods, interaction methods and the technology stack of each web mapping platform, which were compared subsequently.

**TABLE 1:** Name, URL and a basic description of the web mapping platforms included in the competitive analysis

Web mapping platform	URL	Basic description (from source)
Global Inventory of Floras and Traits—GIFT	https://gift.uni‐goettingen.de	A global archive of regional plant checklists, floras, plant functional traits
Map of Life	https://mol.org	Geographical Information visualizations describing species distributions worldwide
Gender Atlas of Austria	http://genderatlas.at/	Atlas visualizing data, indicators and information on the realities of women & men in Austria
Ant Maps	https://antmaps.org/	The goal of antmaps.org is to provide an intuitive and efficient framework for professional and amateur myrmecologists to visualize the known distribution of ant species or higher taxon, and to access the underlying records for those data
Allen Coral Atlas	https://allencoralatlas.org/atlas/	The Allen Coral Atlas goal is to take high‐resolution satellite imagery and advanced analytics to map and monitor the world’s coral reefs in unprecedented detail
Atlas of Switzerland	http://atlasderschweiz.ch/portfolio/aos‐online/	The Atlas of Switzerland Online is a complete atlas framework covering a wide range of categories
Atlas of Biodiversity Conservation in the Coral Triangle	www.marine.auckland.ac.nz/CTMAPS	The atlas of the Coral Triangle showcases all of the currently available marine biodiversity conservation data for the Coral Triangle region
Fish Atlas of Germany and Austria	fischfauna‐online.de	An atlas of fish species in Germany and Austria

Representation is described as the way the information on the map is encoded and here assessed by the presence or absence of 12 categories. The (1) visualizing time, (2) use of animations, (3) use of choropleth maps (i.e. thematic maps where predetermined geographical areas are coloured in proportional steps based on a specific variable), (4) use of graduated symbol maps, (5) use of dot distribution maps, (6) use of heat maps, (7) use of legends, (8) use of landing pages, (9) use of help section, (10) emphasis on visualizations, (11) use of non‐map visualizations (e.g. bar graphs) and (12) use of non‐mercator projections.

Interaction is defined as the ways a user can manipulate the map. Interaction types were selected based on the list of 11 categories provided in Roth et al. (2013) and included the following: (1) arrange/linked views, (2) reexpress, (3) resymbolize, (4) overlay, (5) reproject, (6) pan, (7) zoom, (8) filter, (9) search, (10) retrieve and (11) change basemap.

The technology stack was compared to identify which engines the different atlases and web mapping platforms are using to develop their systems. More specifically, we looked at which mapping libraries are used, and assessed two categories: (1) if a database is used for storing the data and (2) if a download data button or section is included in the interface.

Following the results of the competitive analysis, concrete sketches of the atlas designs or graphical user interface (GUI) were created and a wireframe (Roth et al., 2017) was developed to better visualize the overall structure of the atlas on screen (Figure S1).

### 2.4 Skeleton stage

The skeleton stage involves the arrangement of data objects into meaningful categories and the design of the overall structure and display of the atlas (e.g. the map display window, the sidebar menu and the pop‐up windows).

To better structure the users' interactions with the data, the Atlas of Plant Invasions was divided into three views: world view, continent view and plant view. The views would be accessible through a homepage, functioning like a table of contents. The decision was made to better structure the users' interaction with the data and to address the issue of geographical scale following the visual information seeking mantra ‘Overview first, zoom and filter, then details‐on‐demand’. As the dataset is large, it also gives the user the ability to filter out the data they do not need.

The wireframe prototype created in this stage was sent out as a user test (Figures S2 and S3) to the target user group (including 10 people from the GloNAF core team and nine randomly chosen people from a cartographic background). It tested the utility and usability of the atlas while also collecting input and feedback. The utility questions assessed the ability of the atlas to be a resource for GloNAF‐related questions, while the usability questions asked about ease of use and learnability of the views. The final questions collected feedback regarding subjective satisfaction with the prototype.

### 2.5 Surface stage

The surface stage is arguably the most important stage of the framework. This stage focuses on bringing together the actual design of the map user interfaces and incorporating all map contents to finalize the atlas.

During the surface stage, the visual layout was finalized and the user experience results from the user test during the skeleton stage were implemented. Surface‐level design features, such as icons, buttons and hyperlinks were finalized, along with data content symbology and colour. User feedback also shaped a redesign of the Plant View, which changed the symbology used for the final product. The representation methods, choropleth maps and graduated symbol methods, received positive feedback.

## 3 RESULTS

### 3.1 The GloNAF atlas of plant invasions

**FIGURE 2:**
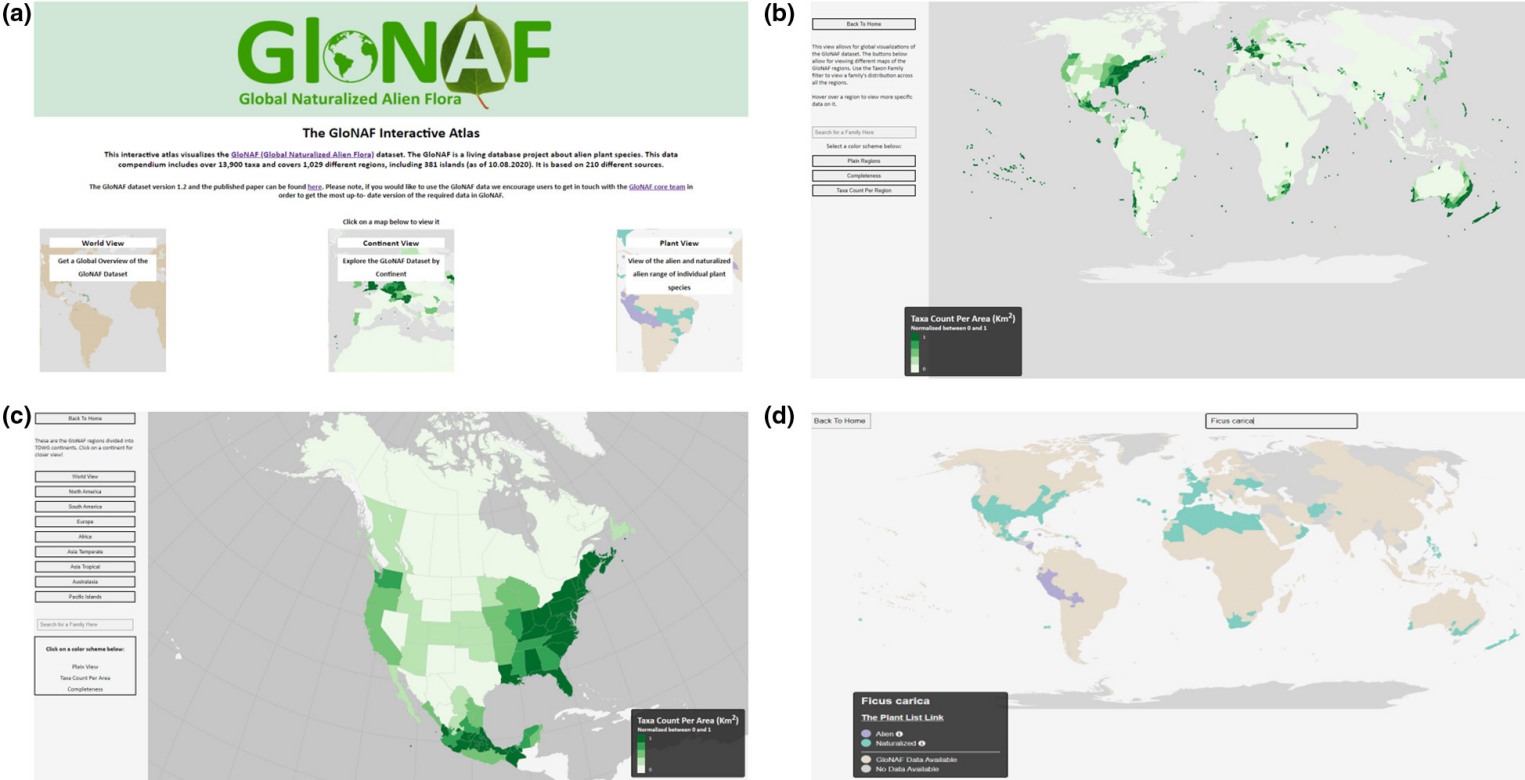
Overview of the atlas of plant invasions interface. (a) Welcome page providing the different view types of the data. (b) The world view showing the taxa count visualization. Shown is the number of naturalized alien vascular plant species, normalized between 0 and 1, per region area. (c) The continent view showing the north American continent. Shown is the number of naturalized alien vascular plant species, normalized between 0 and 1, per region area. (d) The plant view for *Ficus carica*. Shown are regions with naturalized occurrence of the species (‘naturalized’), and regions, where the species is alien, but the invasion status is unknown (‘alien’)

Following our developed framework, the final GloNAF Atlas of Plant Invasions consists of a start homepage that provides an overview on the possible views (Figure 2a) and three views that arrange the atlas objects into meaningful categories: world view, continent view and plant view. Segmentation of the data was crucial to address the issue of geographical scale, which is a unique characteristic of spatial data that makes it different from other kinds of data (MacEachren & Kraak, 2001). For the GloNAF dataset, this geographical separation is crucial for visualizing invasion patterns at different spatial scales, facilitating the identification of scale‐dependent phenomena and patterns (an example for the world view is given in Figure 2b and for the continental view in Figure 2c).

Each view uses the Robinson projection, a compromise projection that balances geographical accuracy with aesthetics. Equal area projections are then used for each continent view. Finally, the plant view is used to visualize the spatial distribution of specific plant taxa. The user can search for particular taxa and is provided with a global map highlighting the regions where the taxon is naturalized (an example for the plant view of *Ficus carica* is given in Figure 2d).

**FIGURE 3:**
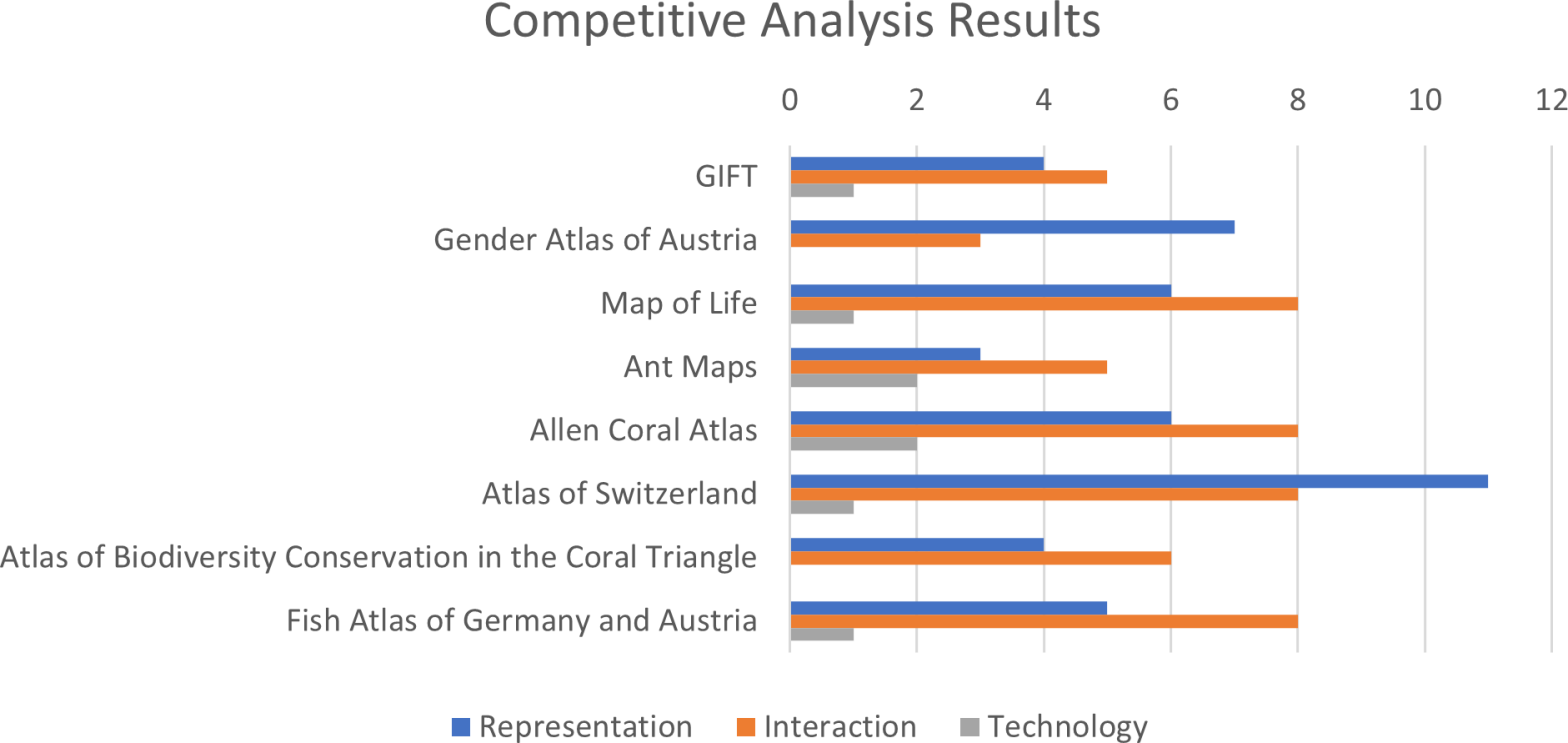
Result from the competitive analysis. Bars indicate the numbers of interaction (maximum of 12 categories), representation (maximum of 11 categories) and technology (maximum of 2 categories) methods used in each atlas or web mapping platform

The results of the three comparisons from the competitive analysis are shown in Figure 3, where we display the cumulative number of available features of the interaction, representation and technology methods for each project. It is, however, important to state that low scores in any of the methods do not necessarily correlate with a low‐quality atlas or web mapping platform. One reason for this is that the competitive analysis does not consider the target audience of each project or the specific needs and goals it is trying to reach but aims to identify key features in comparable applications and to serve as a brainstorming exercise.

In general, the results show that choropleth maps (i.e. thematic maps where predetermined geographical areas are coloured in proportional steps based on a specific variable) are the most popular map type (used in seven online atlases) but graduated symbol maps (used in two online atlases) and dot distribution maps exist as well (used in three online atlases). The interaction results show most GIS‐type interactions such as re‐expression, re‐symbolization and re‐projection are mostly not available (not used in any of the online atlases). On the other hand, more basic interaction methods like panning (used in seven online atlases) and zooming (used in eight online atlases) are included in almost all web mapping platforms, and all projects allow for the ability to change the base map to different styles. Finally, one notable result from the competitive analysis was the diversity of web mapping libraries used. Overall, among the eight online atlases, eight different libraries were used. This demonstrates that there are many current web mapping technologies available that can create an interactive web atlas.

## 4 DISCUSSION

The proposed Combined Atlas Framework provides a conceptual approach to developing an interactive web atlas and offers multiple methods to achieve interface success at each stage of the interactive atlas development process. By combining the five‐stage UCD approach with the three U approach of interface success, we integrated two existing theoretical concepts that complement each other and increase the robustness of the development process by adding an iterative feedback element. This iterative feedback process between the user and the developer is vital to ensure that all relevant facets are integrated in the atlas, and the provision of intermediate products to the target groups strongly contributes to refining the final product along the process (Roth, Quinn, et al., 2015).

Each atlas and web mapping process is unique because it caters to the specific needs of the defined user group. In the Atlas of Plant Invasions, the user group was a team of scientists from the field of invasion science. For catering to their needs, the atlas was designed using the D3 visualization library instead of a tile‐based mapping library. Along with its ability to handle large amounts of spatial data from multiple data sources (e.g. GeoJSON, CSV, JSON), D3 allows for better cartographic representation compared to other mapping libraries because it allows the use of multiple map projections. Other cartographic libraries such as Leaflet, Mapbox and OpenLayers were explored as they provide an easier implementation of usability functions, such as panning, zooming and the ability to use tile maps. Tile‐based technologies allow the developer to facilitate a higher level of exploration with less code but were deemed not adequate for the needs of the Atlas of Plant Invasions at this point in time. Future work might include exploring tile‐based JavaScript libraries and especially if the addition of more complementary data (e.g. land‐use, transportation networks, climatic data layers or Global Biodiversity Information Facility occurrence data) served in tiles from Web Map Services and Web Feature Services.

Another option to further develop the Atlas of Plant Invasions would be the use of server‐side technologies, which usually means using a database such as PostgreSQL. For spatial data, an additional plugin, PostGIS, is often used. Combining PostgreSQL with PostGIS would enable the support of additional geographical objects. PostgreSQL and PostGIS also allow for different data types further increasing the flexibility of the atlas. Finally, storing data in such a database facilitates data maintenance and management. Updating and editing data can be completed in the database itself, and files do not need to be changed.

Following the currently implemented approach using GeoJSONs, each time the data were updated, a new GeoJSON had to be created. This does pose possible issues for future users, as each time the dataset is updated, the files will have to be updated individually. However, by not using server‐side technologies, the interactive atlas can be hosted on platforms like GitHub, which is a well‐known version‐control hosting platform for software development. By hosting the code on GitHub, a URL can be created for free. Another benefit is that the development code for the atlas is available online to be viewed and accessed by anyone supporting FAIR (findable, accessible, interoperable and reusable) data and code policies.

Overall, the Combined Atlas Framework provides an innovative way of developing interactive web mapping products for biodiversity data. The framework further provides developers and scientists with a tool that makes the development process as flexible and transparent as possible to create a product that captures all features and dimensions that are required from the user perspective. The concept of including the user along each step of the development of a web mapping application has been considered in other approaches before (Padilla‐Ruiz et al., 2019; Robinson et al., 2005) and builds the foundation of our Combined Atlas Framework. However, rather than getting user input only after key decisions have been made by the developer, our framework advocates for a closer collaboration and interaction between developers and users. This will not only ensure the adequate focus of the final product, but also reduce the necessity for time‐consuming updates. This assumption is in contrast to other approaches, where early interaction with stakeholders (i.e. target users) has been avoided to provide a more polished product for the first presentation (Slocum et al., 2004). However, such practice only supports the belief that interacting with the target audience using an ‘imperfect’ product has no benefit, whereas on the contrary, these interactions are highly valuable for the development success.

The biodiversity community will greatly benefit from more web mapping applications on the vast amount of mobilized biodiversity data that explicitly consider the relevant target audience. The Combined Atlas Framework is one tool to increase the utility–usability trade‐off of such new web mapping applications by considering the background, expertise and knowledge of the audience it wants to address.

## CONFLICT OF INTEREST

The authors have no conflict of interest to declare.

## AUTHORS’ CONTRIBUTIONS

S.C.H., G.G., F.E. and B.L. conceived the idea, S.C.H. developed the code, S.C.H. and B.L. led the writing with substantial input by G.G., M.J.‐K. and F.E. GloNAF core team members (W.D., F.E., H.K., J.P., P.P., M.K., P.W. and M.W.) built the Global Naturalized Alien Flora database that was used to test the concept. All authors provided valuable feedback during the development of the Atlas of Plant Invasions and provided feedback on the manuscript.

### PEER REVIEW

The peer review history for this article is available at https://publons.com/publon/10.1111/2041‐210X.13820

## Supporting information


Data S1

## Data Availability

The source code for the interactive atlas is freely available online (Sebastian Hancock, 2022), along with the data used at https://github.com/sebastian‐ch/glonafAtlas.

## References

[mee313820-bib-0001] Agafonkin, V. (2010). Leaflet—A JavaScript library for interactive maps. https://leafletjs.com/.

[mee313820-bib-0002] Allen Coral Atlas (2020). Imagery, maps and monitoring of the world’s tropical coral reefs. 10.5281/zenodo.3833242.

[mee313820-bib-0003] Alonso, P. G. (1968). The first atlases. Cartographica, 5, 108–121. 10.3138/W635-647V-3684-11V4

[mee313820-bib-0004] Asaad, I. , Lundquist, C. J. , Erdmann, M. V. , & Costello, M. J. (2019). An interactive atlas for marine biodiversity conservation in the coral triangle. Earth System Science Data, 11, 163–174. 10.5194/essd-11-163-2019

[mee313820-bib-0005] Blackburn, T. M. , Pyšek, P. , Bacher, S. , Carlton, J. T. , Duncan, R. P. , Jarošík, V. , Wilson, J. R. U. , & Richardson, D. M. (2011). A proposed unified framework for biological invasions. Trends in Ecology and Evolution, 26, 333–339. 10.1016/j.tree.2011.03.023 21601306

[mee313820-bib-0006] Bostock, M. , Ogievetsky, V. , & Heer, J. (2011). D^3^ data‐driven documents. IEEE Transactions on Visualization and Computer Graphics, 17, 2301–2309. 10.1109/TVCG.2011.185 22034350

[mee313820-bib-0007] Brummitt, R. K. (2001). World geographical scheme for recording plant distributions (2nd ed.). Carnegie Mellon University.

[mee313820-bib-0008] Brunken, H. & Vatterrott, H.‐R. (2020): Fish species atlas of Germany and Austria.

[mee313820-bib-0009] Capinha, C. , Seebens, H. , Cassey, P. , García‐Díaz, P. , Lenzner, B. , Mang, T. , Moser, D. , Pyšek, P. , Rödder, D. , Scalera, R. , Winter, M. , Dullinger, S. , & Essl, F. (2017). Diversity, biogeography and the global flows of alien amphibians and reptiles. Diversity and Distributions, 23, 1313–1323. 10.1111/ddi.12617

[mee313820-bib-0010] Dawson, W. , Moser, D. , van Kleunen, M. , Kreft, H. , Pergl, J. , Pyšek, P. , Weigelt, P. , Winter, M. , Lenzner, B. , Blackburn, T. M. , Dyer, E. E. , Cassey, P. , Scrivens, S. L. , Economo, E. P. , Guénard, B. , Capinha, C. , Seebens, H. , García‐Díaz, P. , Nentwig, W. , … Essl, F. (2017). Global hotspots and correlates of alien species richness across taxonomic groups. Nature Ecology & Evolution, 1, 0186.

[mee313820-bib-0011] Dornelas, M. , Antão, L. H. , Moyes, F. , Bates, A. E. , Magurran, A. E. , Adam, D. , Akhmetzhanova, A. A. , Appeltans, W. , Arcos, J. M. , Arnold, H. , Ayyappan, N. , Badihi, G. , Baird, A. H. , Barbosa, M. , Barreto, T. E. , Bässler, C. , Bellgrove, A. , Belmaker, J. , Benedetti‐Cechi, L. , … Zettler, M. L. (2018). BioTIME: A database of biodiversity time series for the Anthropocene. Global Ecology and Biogeography, 27, 760–786. 10.1111/geb.12729 30147447 PMC6099392

[mee313820-bib-0012] Dyer, E. E. , Cassey, P. , Redding, D. W. , Collen, B. , Franks, V. , Gaston, K. , Jones, K. E. , Kark, S. , Orme, C. D. L. , & Blackburn, T. M. (2017). The global distribution and drivers of alien bird species richness. PLoS Biology, 15, 1–25. 10.1371/journal.pbio.2000942 PMC5230740 28081142

[mee313820-bib-0013] Dyer, E. E. , Redding, D. W. , & Blackburn, T. M. (2017). The global avian invasions atlas – A database of alien bird distributions worldwide. Scientific Data, 4, 170041. 10.1038/sdata.2017.41 28350387 PMC5369319

[mee313820-bib-0014] Farkas, G. (2017). Applicability of open‐source web mapping libraries for building massive web GIS clients. Journal of Geographical Systems, 19, 273–295. 10.1007/s10109-017-0248-z

[mee313820-bib-0016] Haklay, M. , & Zafiri, A. (2008). Usability engineering for GIS: Learning from a screenshot. The Cartographic Journal, 45, 87–97. 10.1179/174327708X305085

[mee313820-bib-0017] Hancock, S . (2022). Sebastian‐ch/glonafAtlas: GloNAF atlas (v1.0). Zenodo. 10.5281/zenodo.5942103

[mee313820-bib-0018] Hudson, L. N. , Newbold, T. , Contu, S. , Hill, S. L. L. , Lysenko, I. , De Palma, A. , Phillips, H. R. P. , Alhusseini, T. I. , Bedford, F. E. , Bennett, D. J. , Booth, H. , Burton, V. J. , Chng, C. W. T. , Choimes, A. , Correia, D. L. P. , Day, J. , Echeverría‐Londoño, S. , Emerson, S. R. , Gao, D. , … Purvis, A. (2017). The database of the PREDICTS (projecting responses of ecological diversity in changing terrestrial systems) project. Ecology and Evolution, 7, 145–188. 10.1002/ece3.2579 28070282 PMC5215197

[mee313820-bib-0019] Janicki, J. , Narula, N. , Ziegler, M. , Guénard, B. , & Economo, E. P. (2016). Visualizing and interacting with large‐volume biodiversity data using client–server web‐mapping applications: The design and implementation of antmaps.org. Ecological Informatics, 32, 185–193. 10.1016/j.ecoinf.2016.02.006

[mee313820-bib-0020] Jetz, W. , McPherson, J. M. , & Guralnick, R. P. (2012). Integrating biodiversity distribution knowledge: Toward a global map of life. Trends in Ecology and Evolution, 27, 151–159. 10.1016/j.tree.2011.09.007 22019413

[mee313820-bib-0021] MacEachren, A. M. , & Kraak, M.‐J. (2001). Research challenges in geovisualization. Cartography and Geographic Information Science, 28, 3–12. 10.1559/152304001782173970

[mee313820-bib-0022] Machwitz, M. , Hass, E. , Junk, J. , Udelhoven, T. , & Schlerf, M. (2019). CropGIS – A web application for the spatial and temporal visualization of past, present and future crop biomass development. Computers and Electronics in Agriculture, 161, 185–193. 10.1016/j.compag.2018.04.026

[mee313820-bib-0023] Ormeling, F. (1995) Atlas information systems. In Atlas Information Systems. Paper presented at the 17th international cartographic conference, Barcelona.

[mee313820-bib-0024] Padilla‐Ruiz, M. , Stefanakis, E. , & Church, I. (2019). Development of a user‐centered web‐mapping application of ocean Modellers. Marine Geodesy, 42, 507–534. 10.1080/01490419.2019.16666758,

[mee313820-bib-0025] Pagad, S. , Genovesi, P. , Carnevali, L. , Schigel, D. , & McGeoch, M. A. (2018). Introducing the global register of introduced and invasive species. Scientific Data, 5, 1–12. 10.1038/sdata.2017.202 29360103 PMC5779068

[mee313820-bib-0026] Panchaud, N. , Iosifescu‐Enescu, I. , Eichenberger, R. , & Hurni, L. (2013). Service‐driven 3D atlas cartography. Retrieved 5 October 2021 from www.atlasderschweiz.ch/wp‐content/uploads/13_Panchaud_ICC.pdf

[mee313820-bib-0027] Pyšek, P. , Pergl, J. , Essl, F. , Lenzner, B. , Dawson, W. , Kreft, H. , Weigelt, P. , Winter, M. , Kartesz, J. , Nishino, M. , Antonova, L. A. , Barcelona, J. F. , Cabezas, F. J. , Cárdenas, D. , Cárdenas‐Toro, J. , Castaño, N. , Chacón, E. , Chatelain, C. , Dullinger, S. , … van Kleunen, M. (2017). Naturalized alien flora of the world: Species diversity, taxonomic and phylogenetic patterns, geographic distribution and global hotspots of plant invasion. Preslia, 89, 203–274. 10.23855/preslia.2017.203

[mee313820-bib-0028] Richardson, D. M. , Pyšek, P. , Rejmánek, M. , Barbour, M. G. , Panetta, F. D. , & West, C. J. (2000). Naturalization and invasion of alien plants: Concepts and definitions. Diversity & Distributions, 6, 93–107.

[mee313820-bib-0029] Robinson, A. C. , Chen, J. , Lengerich, E. J. , Meyer, H. G. , & MacEachren, A. M. (2005). Combining usability techniques to design Geovisualization tools for epidemiology. Cartography and Geographic Information Science, 32, 243–255. 10.1559/152304005775194700 19960106 PMC2786201

[mee313820-bib-0030] Roth, R. E. , Donohue, R. G. , Sack, C. M. , Wallace, T. R. , & Buckingham, T. M. A. (2013). A process for assessing emergent web mapping technologies. Proceedings of the 26th International Cartographic Conference, 15.

[mee313820-bib-0031] Roth, R. E. , & Harrower, M. (2008). Addressing map Interface usability: Learning from the lakeshore nature preserve interactive map. Cartographic Perspectives, 60, 46–66. 10.14714/CP60.231

[mee313820-bib-0032] Roth, R. E. , Hart, D. , Mead, R. , & Quinn, C. (2017). Wireframing for interactive & web‐based geographic visualization: Designing the NOAA Lake level viewer. Cartography and Geographic Information Science, 44, 338–357. 10.1080/15230406.2016.1171166

[mee313820-bib-0033] Roth, R. E. , Quinn, C. , & Hart, D. (2015). The competitive analysis method for evaluating water level visualization tools. In J. Brus , A. Vondrakova , & V. Vozenilek (Eds.), Modern trends in cartography (pp. 241–256). Springer International Publishing. 10.1007/978-3-319-07926-4_19

[mee313820-bib-0034] Roth, R. E. , Ross, K. , & MacEachren, A. (2015). User‐centered design for interactive maps: A case study in crime analysis. ISPRS International Journal of Geo‐Information, 4(1), 262–301. 10.3390/ijgi4010262

[mee313820-bib-0035] Slocum, T. A. , Sluter, R. , Kessler, F. , & Yoder, S. (2004) A qualitative evaluation of MapTime, A program for exploring spatiotemporal point data. Cartographica, 39, 43–68. https://utpjournals.press. 10.3138/92T3‐T928‐8105‐88X7.

[mee313820-bib-0036] Tsou, M.‐H. (2011). Revisiting web cartography in the United States: The rise of user‐centered design. Cartography and Geographic Information Science, 38(3), 250–257. 10.1559/15230406382250

[mee313820-bib-0037] Tsou, M.‐H. , & Curran, J. M. (2008). User‐centered design approaches for web mapping applications: A case study with USGS hydrological data in the United States. In M. P. Peterson (Ed.), International Perspectives on Maps and the Internet (pp. 301–321). Springer. 10.1007/978-3-540-72029-4_20

[mee313820-bib-0038] van Kleunen, M. , Dawson, W. , Essl, F. , Pergl, J. , Winter, M. , Weber, E. , Kreft, H. , Weigelt, P. , Kartesz, J. , Nishino, M. , Antonova, L. A. , Barcelona, J. F. , Cabezas, F. J. , Cárdenas, D. , Cárdenas‐Toro, J. , Castaño, N. , Chacón, E. , Chatelain, C. , Ebel, A. L. , … Pyšek, P. (2015). Global exchange and accumulation of non‐native plants. Nature, 525, 100–103. 10.1038/nature14910 26287466

[mee313820-bib-0039] van Kleunen, M. , Pyšek, P. , Dawson, W. , Essl, F. , Kreft, H. , Pergl, J. , Weigelt, P. , Stein, A. , Dullinger, S. , König, C. , Lenzner, B. , Maurel, N. , Moser, D. , Seebens, H. , Kartesz, J. , Nishino, M. , Aleksanyan, A. , Ansong, M. , Antonova, L. A. , … Winter, M. (2019). The global naturalized alien Flora (GloNAF) database. Ecology, 100, e02542. 10.1002/ecy.2542) 30341991

[mee313820-bib-0040] Vozenilek, V. (2019). Atlases and systems theory within systematic cartography. Abstracts of the ICA, 1, 1–2. 10.5194/ica-abs-1-386-2019

[mee313820-bib-0041] Weigelt, P. , König, C. , & Kreft, H. (2020). GIFT – A global inventory of floras and traits for macroecology and biogeography. Journal of Biogeography, 47, 16–43. 10.1111/jbi.13623

[mee313820-bib-0042] Wenk, M. , Ledermann, F. , Riegler, M. , Schmidt, M. & Aufhauser, E. (2015). Ein genderATlas für Österreich. http://genderatlas.at/

