# Supplementary material for: Introducing the combined atlas framework for large‐scale web‐based data visualization: The GloNAF atlas of plant invasion
Source: Methods Ecol Evol. 2022 Feb 22;13(5):1073–81. doi: 10.1111/2041-210X.13820 (PMC9305529; doi:10.1111/2041-210X.13820)
Supplement: Supplementary file 1 — Data S1 [file MEE3-13-1073-s001.pdf]

Supplementary Information: Introducing the Combined Atlas Framework for large-scale web-based data visualization – The GloNAF Atlas of Plant Invasion

Sebastian C. Hancock, Franz Essl, Menno-Jan Kraak, Wayne Dawson, Holger Kreft, Petr Pyšek, Jan Pergl, Mark van Kleunen, Patrick Weigelt, Marten Winter , Georg Gartner, Bernd Lenzner

Background information on the five-stage UCD approach

Below we elaborate on the conceptual content of each stage from the five-stage UCD approach.

Strategy stage: This stage involves defining both the target users and their needs, recognizing the differences between the wide range of potential users in the field (Haklay & Zafiri 2008). In consideration of the target user group, there are four axioms that the designers and developers should embrace (Roth et al. 2015b). First, domain experts do not necessarily represent target users, as they often hold more experience and knowledge than the typical user. Second, the target users are unlikely to know what they want when first contacted, meaning that it is the developer's job to translate their requests into tangible functional requirements (i.e., see scope stage of this approach). Third, the target users are likely to evolve over time, and therefore the interface should develop with the target users. Fourth, the target users can be diverse in their ability, expertise, motivation, and knowledge of their domain and interactive map use.

Scope stage: This stage involves translating what the target users want into tangible goals, establishing the scope of the interactive atlas. Tsou and Curran (2008) describe two aspects of this stage: functional mapping specification and map-content requirement.

Function mapping specification refers to identifying the major mapping tasks as determined by the user needs and map objectives, which were defined in stage one. The map content requirement includes the data required for the web mapping platform.

Structure stage: This stage involves the formalization of the mapping functionalities of the atlas by creating a list of tools needed in the atlas. Examples of these functionalities include spatial queries, buffering, and help functions. These functionalities are determined through analysis of the users' needs and the identified scope from stages one and two. Extensive atlas research and competitive analysis of similar thematic web atlases and web maps can help to achieve success at this stage (Nielsen 1992). The competitive analysis method may be especially beneficial when the design and development team know little about the application domain and is considered essential given the pace of technological change (Roth et al. 2015b). We thus recommend the inclusion of a competitive analysis in stage three. Finally, stage three also consists of itemizing the atlas data contents by developing a more formal list of data needed based on stage two.

Skeleton stage: The skeleton stage involves the arrangement of data objects into meaningful categories and the design of the overall structure and display of the atlas (e.g., the map display window, the sidebar menu, and the pop-up windows). Segmentation of the screen surface is essential (Cartwright et al. 1999). For the atlas, the map should always be the main part of the web page. At this stage, a wireframe, i.e., a rough visual outline of a proposed application will be created that can be used as a prototype to collect input and feedback from target users (Lloyd 2009). Prototyping is recognized as essential for incremental improvement of the utility and usability of an application, especially in cartography (Roth et al. 2017).

A second step is spatial layer management, which requires intelligent and thought-out data management of layers that hold different types of information (Cartwright et al.

1999). As the user will be activating and deactivating layers on their own, it is important to consider issues such as opacity, organization, and hidden layers aspects. Opacity is important because if two layers are activated, they should both be visible and correctly display the respective content. Organization involves the layer ordering and knowing which layers load first or appear first in the interface. The order depends on the user interaction and the identified specific needs. Hidden layer aspects deal mostly with click or hover interactions. Thematic information on a region might not appear on a selected layer; however, it still might be required for a click or hover pop-up interaction information window. The option to access underlying layers, or layers not on the top, is important and necessary for successful interaction.

Surface stage: The surface stage is arguably the most important stage of the framework. This stage focuses on bringing together the actual design of the map user interfaces and incorporating all map contents to finalize the atlas. The design of graphic icons, buttons, and window layouts are major parts of this stage. Map symbology, fonts, and color schemes for different map layers are also completed during this stage. When completing the surface stage, it is important to test the visuals on multiple web browsers to ensure compatibility and test the fonts, colors, and usability of the atlas.

One important aspect of these five stages is that their development can be overlapping if necessary. For example, the structure-stage development can be started before the completion of the scope stage. This is useful because if there are major changes in the design structure, those changes can be re-examined immediately at the scope stage and be appropriately modified on the structure stage (Tsou & Curran 2008).

Background information on the three U's of interface success

The three U's of Interface Success for interactive maps as described in Roth et al. (2015a) focus on usability, utility, and the users of a web-mapping application. The different facets are outlined below.

Usability describes the ease of using an interface to complete the user's desired set of objectives (Grinstein et al. 2003). High usability seeks to reduce the time it takes to perform a routine task or limit the number of errors that might occur when solving a specific problem (Robinson et al. 2005). There are five measures of usability listed by Nielsen (1992): learnability, efficiency, memorability, error frequency and severity, and subjective satisfaction.

Utility describes the usefulness of an interface for completing the user's desired set of objectives (Nielsen 1992). Utility can be evaluated by establishing benchmark tasks, or representative combinations of user objectives and information content (Roth et al. 2015a). Ideally, usability and utility are at their maximum capacity via iterative interface refinement and user-task analysis to determine what utility needs are required to accomplish the atlas' goals (Robinson et al. 2011). However, as software complexity increases, usability and utility tend to play out as competing forces introducing a utility-usability trade-off (Robinson et al. 2011), which has gained increased importance in the era of mobile devices. The best way to resolve this trade-off is to seek input from the target user group. Identifying which tasks are important and not important can reduce superfluous functionality and identify missing functionality (Roth et al. 2013).

Defining **users** (i.e., the community of users the atlas is intended to support) is important to understand the initial functional requirements for the interactive atlas (Aditya & Kraak 2005, Roth et al. 2015a). Including the users and their specific needs is fundamental to an

effective and successful product development and is thus applied in many UCD web mapping processes (Robinson et al. 2005, Padilla-Ruiz et al. 2019). Co-creation is important in decision making processes for certain product details at multiple steps throughout the process, and is preferable over obtaining user input only after key decisions have been made by the developers (Slocum et al. 2004). The final product should therefore be based primarily on the needs of the users and not the developers.

The Combined Atlas Framework based on the above outlined theoretical approaches adds an iterative element to the five-stage process to gather feedback on the utility and usability of the atlas. This enables the developer to better measure interface success and increases flexibility of the process. While the user → utility → usability loop is most advantageous between the skeleton and surface stages, it can in practice be integrated at any of the five stages as new information gathered from the users will result in updated utility and usability purposes.

Data challenges during the construction of the Atlas of Plant Invasions

The GloNAF dataset provided some visualization challenges. It includes 1029 geographic regions worldwide, of which 381 are islands (van Kleunen et al. 2019). The regions themselves are not regular, meaning some represent entire countries, while others are for example small islands within an archipelago. Most regions are not overlapping, however, in some regions (e.g. Chile) overlapping regions occur. Cartographic generalization, or the abstraction of pertinent data through reduction and aggregation, was used to simplify these overlapping areas.

Additionally, within the dataset, there are several topographic and spatial quality issues which are heterogeneous in their distribution. For example, figure S4 shows quality issues related to the level of detail of the delineations of regions in the dataset. To avoid distraction of the user by these inconsistencies, zooming was restricted up to a level where they are not noticeable as the level of border detail does not infer with the message conveyed in the atlas.

References

Aditya, T., & Kraak, M. J. (2005). The atlas as a portal for data discovery in the GDI : prospects and development. In *ICC 2005: Proceedings of the 22nd international cartographic conference: mapping approaches into a changing world* (pp. 10). International Cartographic Association.

Cartwright, W., Peterson, M. P., & Gartner, G. (1999). *Multimedia Cartography*. Berlin, Heidelberg: Springer Berlin Heidelberg. https://doi.org/10.1007/978-3-662-03784-3

Grinstein, G., Kobsa, A., Plaisant, C., & Stasko, J. T. (2003). Which comes first, usability or utility? *IEEE Transactions on Ultrasonics, Ferroelectrics and Frequency Control*, 605–606. https://doi.org/10.1109/VISUAL.2003.1250426

Haklay, M., & Zafiri, A. (2008). Usability Engineering for GIS: Learning from a Screenshot. *The Cartographic Journal*, 45, 87–97. https://doi.org/10.1179/174327708X305085

Lloyd, David (2009). Evaluating human-centered approaches for geovisualization. (Unpublished Doctoral thesis, City University London)

Nielsen, J. (1992) The usability engineering life cycle. *Computer*, 25, 12-22. https://doi.org/10.1109/2.121503.

Padilla-Ruiz, M., Stefanakis, E., & Church, I. (2019) Development of a User-Centered Web-Mapping Application of Ocean Modellers. Marine Geodesy, 42, 507-534. https://www.tandofline.com/doi/full/10.1080/01490419.2019.16666758.

Robinson, A. C., Chen, J., Lengerich, E. J., Meyer, H. G., & MacEachren, A. M. (2005). Combining Usability Techniques to Design Geovisualization Tools for Epidemiology. *Cartography and Geographic Information Science*, 32, 243–255. https://doi.org/10.1559/152304005775194700

Robinson, A. C., MacEachren, A. M., & Roth, R. E. (2011). Designing a web-based learning portal for geographic visualization and analysis in public health. *Health Informatics Journal*, 17, 191–208. https://doi.org/10.1177/1460458211409718

Roth, R. E., Donohue, R. G., Sack, C. M., Wallace, T. R., & Buckingham, T. M. A. (2013). A Process for Assessing Emergent Web Mapping Technologies. *Proceedings of the 26th International Cartographic Conference,* 15.

Roth, R. E., Quinn, C., & Hart, D. (2015a). The Competitive Analysis Method for Evaluating Water Level Visualization Tools. In J. Brus, A. Vondrakova, & V. Vozenilek (Eds.), *Modern Trends in Cartography* (pp. 241–256). Springer International Publishing. https://doi.org/10.1007/978-3-319-07926-4_19

Roth, R. E., Ross, K., & MacEachren, A. (2015b). User-Centered Design for Interactive Maps: A Case Study in Crime Analysis. *ISPRS International Journal of Geo-Information*, *4*(1), 262–301. https://doi.org/10.3390/ijgi4010262

Roth, R. E., Hart, D., Mead, R., & Quinn, C. (2017). Wireframing for interactive & web-based geographic visualization: Designing the NOAA Lake Level Viewer. *Cartography and Geographic Information Science*, *44*, 338–357. https://doi.org/10.1080/15230406.2016.1171166

Slocum, T. A., Sluter, R., Kessler, F., & Yoder, S. (2004) A Qualitative Evaluation of MapTime, A Program For Exploring Spatiotemporal Point Data. *Cartographica: The International Journal of Geographic Information and Geovisualization*, 39, 43-68. https://utpjournals.press/doi/10.3138/92T3-T928-8105-88X7.

Tsou, M.-H., & Curran, J. M. (2008). User-Centered Design Approaches for Web Mapping Applications: A Case Study with USGS Hydrological Data in the United States. In M. P. Peterson (Ed.), *International Perspectives on Maps and the Internet* (pp. 301–

321). Springer Berlin Heidelberg. https://doi.org/10.1007/978-3-540-72029-4_20

van Kleunen, M., Pyšek, P., Dawson, W., Essl, F., Kreft, H., Pergl, J., Weigelt, P., Stein, A., Dullinger, S., König, C., Lenzner, B., Maurel, N., Moser, D., Seebens, H., Kartesz, J., Nishino, M., Aleksanyan, A., Ansong, M., Antonova, L. A., Barcelona, J. F., Breckle, S. W., Brundu, G., Cabezas, F. J., Cárdenas, D., Cárdenas-Toro, J., Castaño, N., Chacón, E., Chatelain, C., Conn, B., de Sá Dechoum, M., Dufour-Dror, J.-M., Ebel, A.-L., Figueiredo, E., Fragman-Sapir, O., Fuentes, N., Groom, Q. J., Henderson, L., Inderjit, Jogan, N., Krestov, P., Kupriyanov, A., Masciadri, S., Meerman, J., Morozova, O., Nickrent, D., Nowak, A., Patzelt, A., Pelser, P. B., Shu, W.-S., Thomas, J., Uludag, A., Velayos, M., Verkhosina, A., Villaseñor, J. L., Weber, E., Wieringa, J., Yazlık, A., Zeddam, A., Zykova, E. & Winter M. (2019): The Global Naturalized Alien Flora (GloNAF) database. – *Ecology*, 100, e02542. https://doi.org/10.1002/ecy.2542)

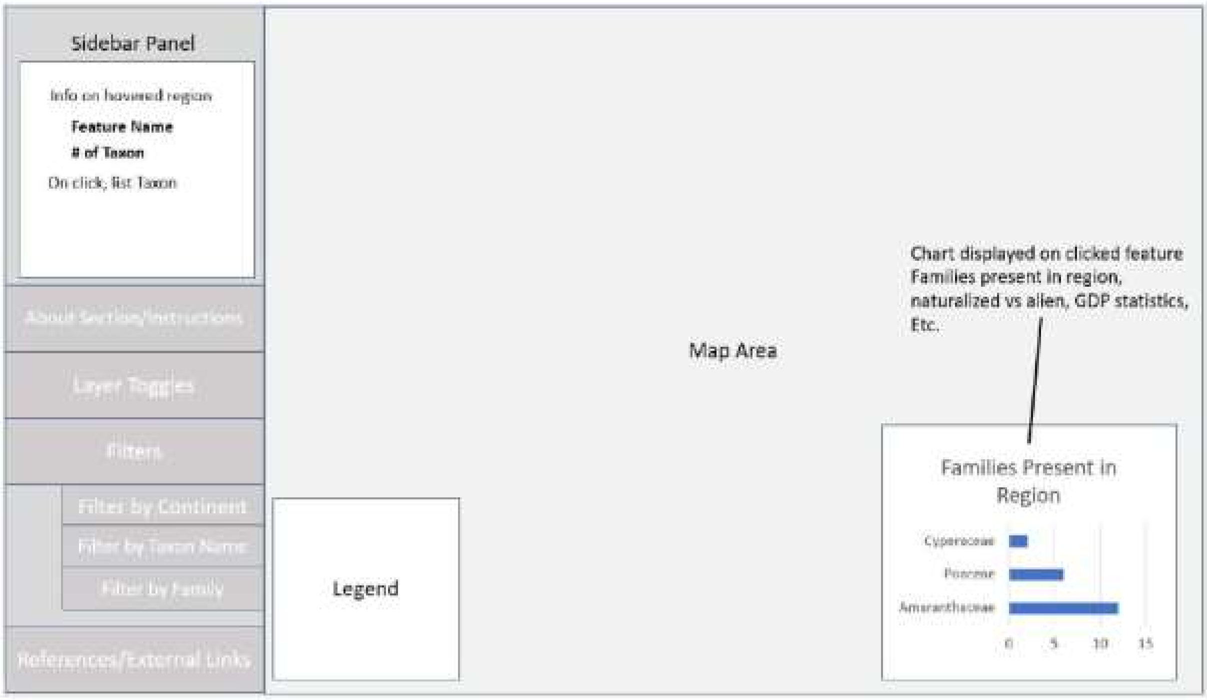

Figure S1: Sketch of the atlas graphical user interface developed in the structure stage. The figure shows the basic wireframe of the atlas views. The view is divided into two main parts: the map area and the sidebar panel. These parts are consistent throughout each map view and the design allows the map area to take up most of the space on the screen. The left-hand sidebar panel contains buttons, help functions, and text information. A legend will be visible in the bottom left of the map area, and popup windows for hovering and click interactive events will appear as well.

These questions measure the utility of the atlas. After taking a moment to familiarize yourself with the atlas, answer the questions however possible.
If the maps are too large for the screen, please let me know in the comments in the end. A workaround for this is to zoom out in your browser.

2. How many Taxa are in Japan?

3. What is the Completeness of California?

- [ ] 1
- [ ] 2
- [ ] 3
- [ ] I don't know

4. For the Solanaceae family, can you name a region with more than 50 members?

These questions measure the utility of the atlas. After taking a moment to familiarize yourself with the atlas, answer the questions however possible.
If the maps are too large for the screen, please let me know in the comments in the end. A workaround for this is to zoom out in your browser.

5. Can you name a region where Solanum melongena is naturalized?

6. Can you name a region where Solanum melongena is alien?

7. On the African continent can you name a region with the highest level of completeness?

8. For the region Florida (in the United States), can you determine the number of members of the Polygonaceae family?

Figure S2: User test sent out on at the skeleton stage based on the developed wireframe. The questions assess the utility of the atlas.

9. How easy was it for you to answer these questions?

○	○	○	○	○
Difficult	Not Easy	Neutral	Moderately Easy	Very Easy

10. Overall, do you find this atlas layout visually pleasing?

○	○	○	○	○
1 – Not Pleasing at all	2	3	4	5 – Very Pleasing

11. Which View was the easiest to use?

- ○ World View
- ○ Plant View
- ○ Continent View

12. Which View was the most visually pleasing?

- ○ World View
- ○ Plant View
- ○ Continent View

13. Which View did you use to answer most of the questions?

- ○ World View
- ○ Plant View
- ○ Continent View
- ○ Even mix of all three

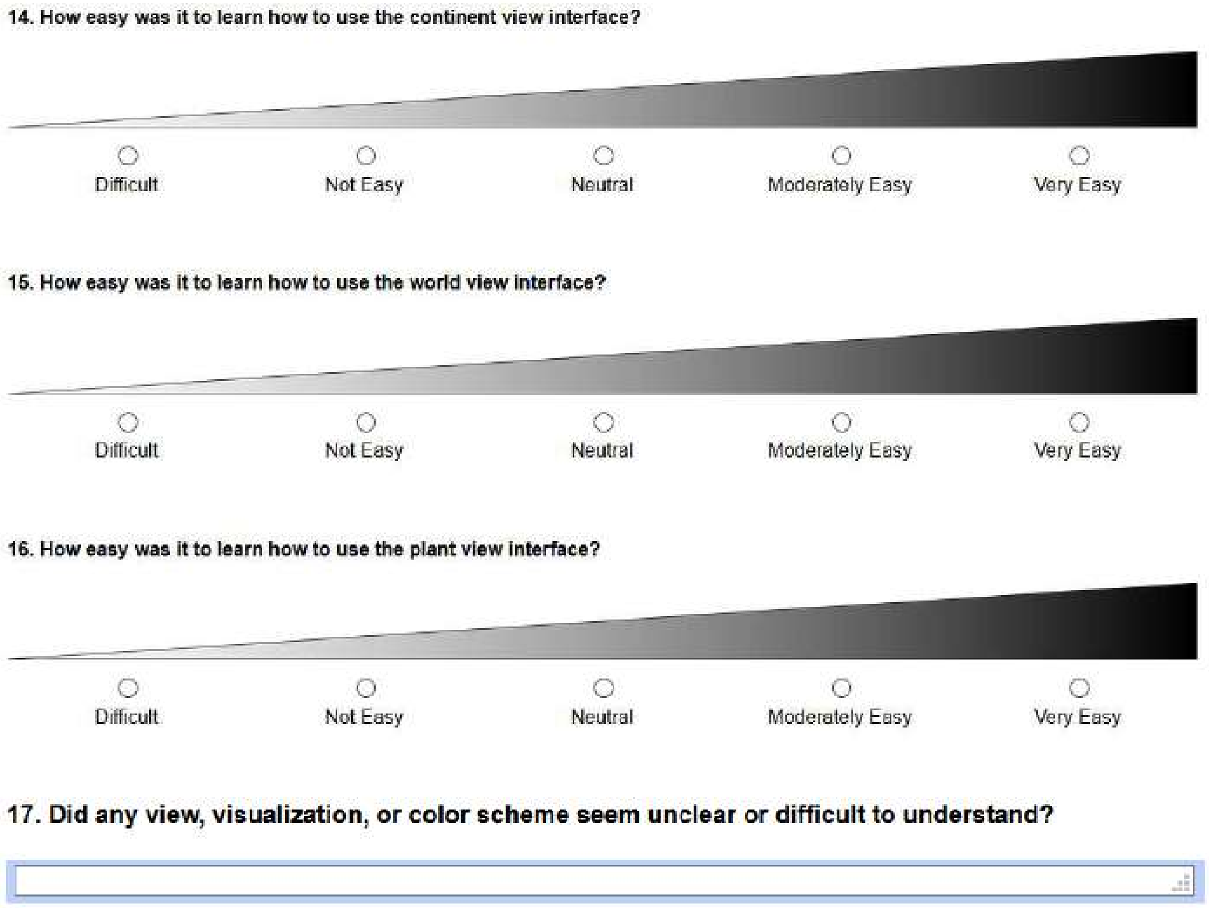
14. How easy was it to learn how to use the continent view interface?

Difficult | Not Easy | Neutral | Moderately Easy | Very Easy

15. How easy was it to learn how to use the world view interface?

Difficult | Not Easy | Neutral | Moderately Easy | Very Easy

16. How easy was it to learn how to use the plant view interface?

Difficult | Not Easy | Neutral | Moderately Easy | Very Easy

17. Did any view, visualization, or color scheme seem unclear or difficult to understand?

Figure S3: User test sent out on at the skeleton stage based on the developed wireframe. The questions assess the usability of the atlas.

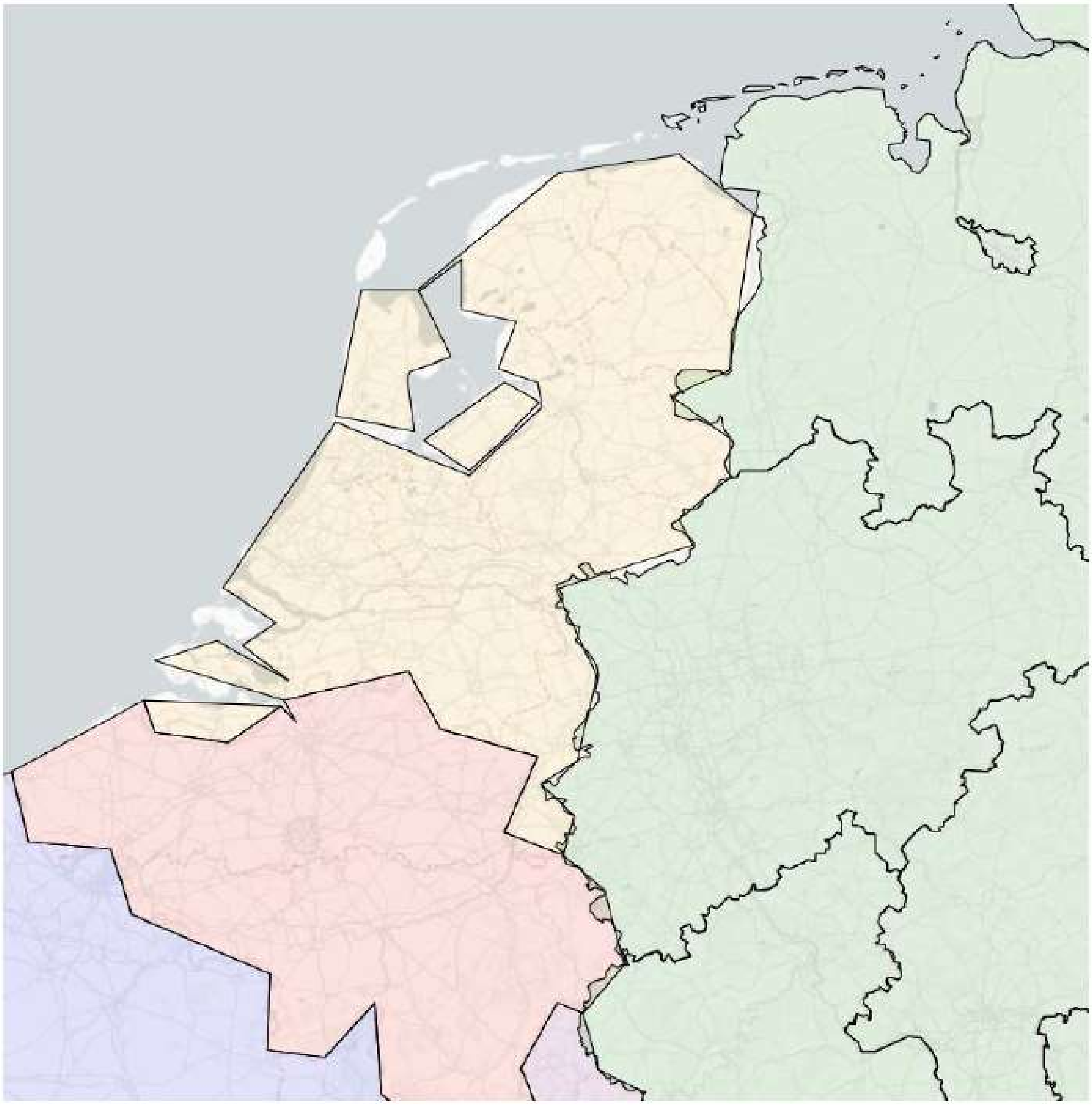

Figure S4: Difference in border quality that is common throughout the GloNAF dataset. The Netherlands (yellow), Belgium (red), France (blue), and Luxemburg (purple) have simplified borders, while German states (green) have more detailed boundaries and do not topographically line up with the others.
